# Intraoperative Cortico-Cortical Evoked Potential Monitoring for Tumor Resection in Eloquent Regions: A Systematic Review

**DOI:** 10.7759/cureus.91923

**Published:** 2025-09-09

**Authors:** Muhammad Idrees, Muhammad M Malhi, Muhammad Haider, Maham Mujeeb, Maryum Tayyab, Hamza Mahmood, M Mohsin

**Affiliations:** 1 Geriatric Medicine, Walsall Manor Hospital, Walsall, GBR; 2 Acute Medicine, Eastbourne District General Hospital, Eastbourne, GBR; 3 General Practice, National Health Service Health Education England, London, GBR; 4 Acute Medicine, Walsall Manor Hospital, Walsall, GBR; 5 Cardiology, Eastbourne District General Hospital, Eastbourne, GBR; 6 Neurosurgery, Faisalabad Medical University, Faisalabad, PAK; 7 Internal Medicine, Allama Iqbal Medical College, Lahore, PAK

**Keywords:** brain tumor surgery, ccep, eloquent cortex, functional mapping, intraoperative monitoring

## Abstract

Preserving neurological function during brain tumor surgery near the eloquent cortex is a major challenge. Cortico-cortical evoked potentials (CCEPs) have emerged as a promising modality for intraoperative mapping of functional connectivity, especially in areas responsible for language, movement, and sensory integration. Unlike traditional motor or sensory evoked potentials, CCEPs allow assessment of bidirectional cortico-cortical pathways and have shown utility in epilepsy surgery. Their integration into tumor resections near eloquent areas remains a developing frontier. This systematic review aimed to evaluate the clinical utility, methodological consistency, and limitations of CCEP monitoring during brain tumor surgery involving eloquent regions. The systematic search of the literature occurred in PubMed, Scopus, Web of Science, and Google Scholar, with the search period between 2015 and 2024. Eight articles were deemed eligible for inclusion, presenting original data on the use of CCEP intraoperatively or more antecedently in surgical settings involving the eloquent cortex. The extraction was based on the demographic information of the patient, stimulation parameters, signal processing, and clinical response. The risk of bias was evaluated through ROBINS-I (Risk of Bias in Non-randomized Studies of Interventions). CCEPs were associated with improved mapping accuracy in six of eight studies and enabled enhanced visualization of cortico-cortical connectivity. Studies also highlighted variability in stimulation protocols and the influence of signal preprocessing. Only three studies showed a low overall risk of bias, reflecting heterogeneity in design and population. CCEP monitoring is a promising tool for functional mapping during tumor resection in the eloquent cortex. However, methodological inconsistencies and limited tumor-specific data highlight the need for standardized protocols and larger, prospective studies.

## Introduction and background

The preservation of neurological function during brain tumor surgery, particularly within the eloquent cortex, remains a principal challenge in neurosurgical oncology [1]. Eloquent areas, responsible for speech, movement, and sensory integration, are functionally diverse and anatomically variable, often displaced or infiltrated by neoplastic lesions [2]. Advances in neuroimaging, intraoperative stimulation, and real-time electrophysiology have significantly enhanced the neurosurgeon’s capacity to balance maximal tumor resection with functional preservation [3,4]. Cortico-cortical evoked potentials (CCEPs) are one of them, and they appeared to be a potential intraoperative and extraoperative functional connectivity mapping tool [5].

By applying single-pulse electrical stimulation (SPES) to a cortical location and obtaining an evoked response in recordings at anatomically far, but functionally connected, sites, CCEPs are produced [6]. This method takes advantage of the inherent structure of brain networks, and it can gain insights into the white matter connection in real-time that conventional tractography could not [7]. Compared to motor evoked potentials (MEPs), or somatosensory evoked potentials (SSEPs), which are activities based on long-range afferent or efferent processing, CCEPs can demonstrate bilateral cortico-cortical pathways-which can be especially useful in language and association cortices [8].

While CCEPs have been widely applied in epilepsy surgery to delineate seizure propagation pathways, their application in tumor resection near eloquent areas is comparatively recent [9]. Early studies suggest that CCEPs may enhance the precision of awake craniotomies, supplement diffusion-based tractography, and inform decisions about safe resection margins [10]. Yet, significant variability in stimulation parameters, neurophysiological assessment techniques, and clinical endpoints remains a barrier to widespread adoption [11,12]. Additionally, the influence of pathological substrates (such as tumor-related edema, gliosis, or altered cortical excitability) on CCEP reliability is not fully understood [13].

Existing literature on CCEP use in tumor surgery is scattered across case series, technical reports, and small cohort studies [14]. To date, no comprehensive synthesis has been performed to consolidate findings, identify methodological trends, or assess clinical relevance [15]. Given the rapid evolution of neurophysiological mapping techniques and the pressing need for precision-guided surgery in the functional cortex, a systematic review of this domain is timely and necessary.

The present review aims to systematically evaluate the role of CCEP monitoring in tumor surgeries involving eloquent regions [16]. By analyzing study design, stimulation protocols, electrophysiological monitoring methods, and clinical outcomes, we seek to highlight both the utility and limitations of CCEP in contemporary neurosurgical practice and to inform future directions for standardization and integration.

## Review

Methodology

This systematic review has been carried out and was compliant with PRISMA 2020 (Preferred Reporting Items for Systematic Reviews and Meta-Analyses) standards, ensuring methodological transparency and replicability. The main objective was to assess the application and usefulness of CCEP, monitoring of brain tumor surgery in the eloquent brain areas in a comprehensive manner.

Search Strategy and Data Sources

The systematic literature search was conducted in four electronic databases (PubMed, Scopus, Web of Science, Google Scholar) on the studies that were published between January 2015 and December 2024. A combination of Medical Subject Headings (MeSH) and keywords, including CCEP, cortico-cortical evoked potentials, intraoperative monitoring, brain tumor surgery, eloquent cortex, and functional mapping, was used to conduct the search. Reference lists of considered articles and reviews were manually searched as well to find other pertinent literature.

Eligibility Criteria

Only studies that provided original information on the utilization of CCEP monitoring in surgeries on brain tumors or epilepsy in terms of eloquent cortex areas were included in the search. Studies included had to either detail intraoperative or preoperative CCEP techniques, or provide pertinent outcome measures, including mapping accuracy, surgical effectiveness, or the like, elucidation of cortico-cortical connection patterns. Considered were peer-reviewed articles, published in English only. Studies showing animal studies, editorials, commentaries, or case reports were excluded. Moreover, the research that did not provide primary CCEP data or did not outline their methodology clearly was not included in the review.

Study Selection and Data Extraction

Screening was undertaken by two independent reviewers in chronological order, starting with title and abstract screening, followed by a full-text screen of potentially eligible articles. Any conflicts between reviewers would be solved by discussion or possible adjudication of a third reviewer where necessary. The data were extracted using a standard form to isolate critically important information, such as study metadata (author, year of publication, country), patient description and diagnosis, CCEP methodology (stimulation modality and parameters), analytical model, and important clinical or methodological findings.

Synthesis Strategy

Given the considerable heterogeneity observed across the included studies-particularly in terms of CCEP protocols, patient populations, and outcome measures, a meta-analytic approach was deemed inappropriate. Instead, a structured narrative synthesis was conducted. This synthesis incorporated vote counting to evaluate the consistency and directionality of reported effects, alongside descriptive statistics to summarize sample sizes and methodological details. Thematic analysis was also employed to organize findings into key clinical and methodological domains. Visual tabulation was used to present study characteristics, risk of bias assessments, and outcome classifications for comparative interpretation.

Risk of Bias Assessment

In the case of observational studies, the ROBINS-I (Risk of Bias in Non-randomized Studies of Interventions) instrument was applied [13]. When there were no randomized controlled trials (RCTs), the suitable RoB 2.0 adapted criteria were used [14]. The risk assessment was performed in seven areas, such as confounding, selection bias, intervention classification, deviations, missing data, measurement, and selective reporting. Overall, three studies demonstrated a low risk of bias, while the remaining exhibited moderate to serious concerns, particularly in the domains of confounding and selection bias. Variability in stimulation protocols and outcome reporting further contributed to inconsistency across studies. Additionally, small sample sizes and a lack of standardized follow-up limited the generalizability of findings. Publication bias could not be ruled out due to the predominance of single-center reports and technical case series. These methodological shortcomings highlight the need for well-designed, multicenter prospective trials with standardized CCEP monitoring protocols to strengthen the evidence base.

Assessment of Bias and Heterogeneity

As fewer than 10 studies were eligible (n=8), funnel plot asymmetry and Egger’s regression were not conducted due to reduced interpretability. Additionally, Cochran’s Q and I² statistics were not calculated as meta-analytic synthesis was not pursued. Instead, heterogeneity was described qualitatively.

Results

Study Selection

**Figure 1:**
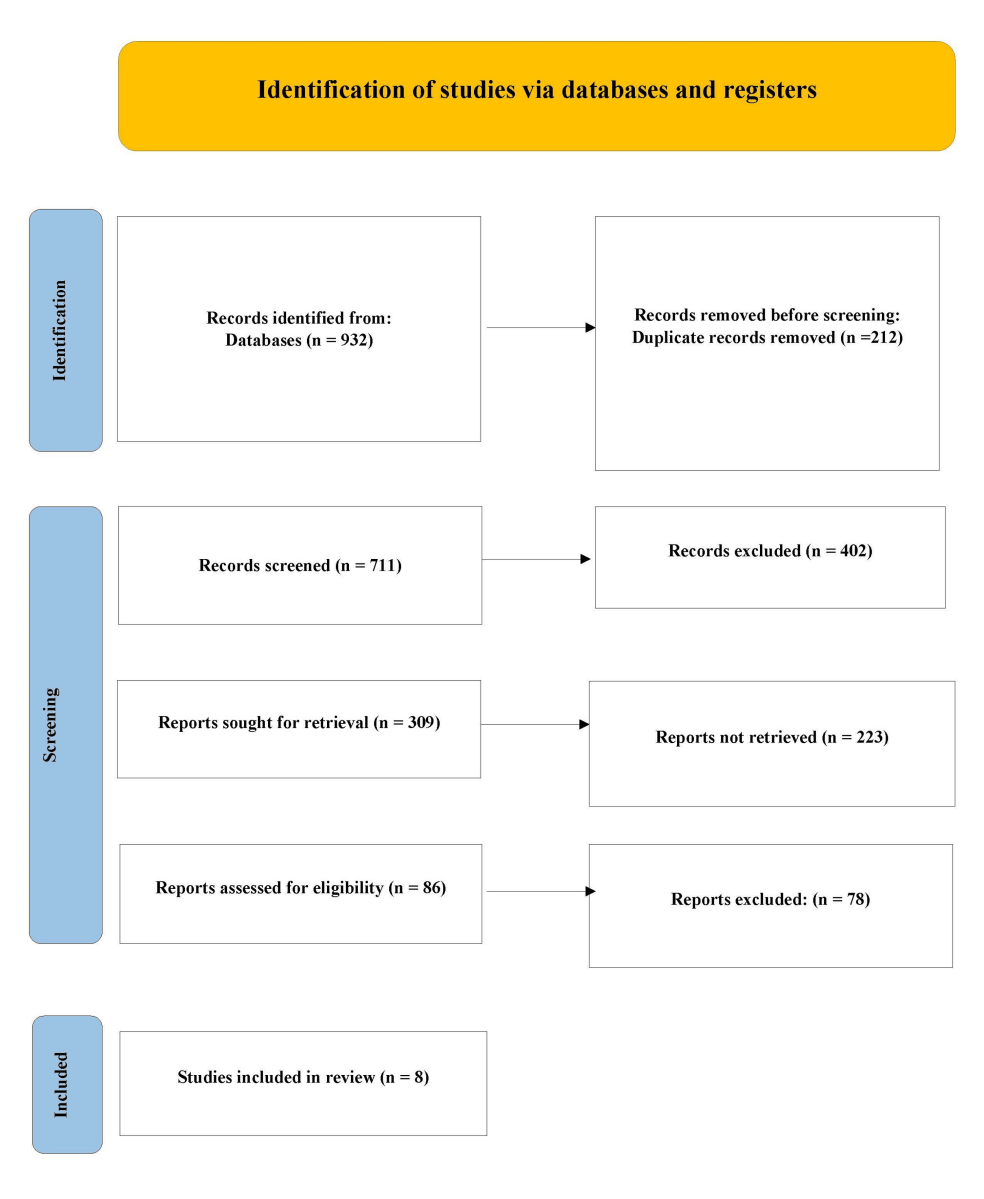
PRISMA flow diagram PRISMA: Preferred Reporting Items for Systematic Reviews and Meta-Analyses

A total of 923 records were retrieved. After removing 212 duplicates, 711 abstracts were screened. Thirty-five full texts were evaluated, and 8 studies fulfilled the inclusion criteria. The study selection process is visualized in Figure 1.

Table 1 outlines the core characteristics of the eight studies included in this review. It summarizes key details such as the study authors, country of origin, patient populations, surgical context, the CCEP techniques employed, and major findings. This table provides essential context for understanding the diversity in methodologies and clinical objectives across the literature. 

**Table 1:** Characteristics of included studies SEEG: stereo-electroencephalography; ECoG: electrocorticography; PCA: principal component analysis; VCP: volume-conducted potential; DTI: diffusion tensor imaging; DES: direct electrical stimulation; EP: evoked potential; CCEP: cortico-cortical evoked potential; SOZ: seizure onset zone

Serial no.	Author (year)	Country	Study population	Surgical context	CCEP technique	Key findings
1	Lega et al. (2015) [15]	USA	Epilepsy (n=37)	SEEG	Mapping ictal spread	Higher coherence in early spread
2	Shimada et al. (2017) [16]	Japan	Epilepsy (n=8)	ECoG	Volume potential analysis	PCA reveals VCP contamination
3	Prime et al. (2018) [17]	Australia	N/A	SEEG	Protocol overview	Summarized safety guidance
4	Chirchiglia et al. (2020) [18]	Italy	Tumor	DTI tractography	Opinion-based	Promoted multimodal imaging
5	Boyer et al. (2021) [19]	France	Tumor (n=2)	Awake surgery	DES-EP	Differentiated EP types
6	Seidel et al. (2022) [20]	Germany	Tumor	Intraop mapping	DES+CCEP	Cortical/subcortical mapping
7	Hays et al. (2023) [21]	USA	Epilepsy (n=15)	SEEG	SPES titration	Higher N1 enhances SOZ detection
8	Levinson et al. (2024) [22]	USA	Mixed	ECoG	Signal processing	Filters impact CCEP integrity

To assess the consistency of study outcomes, Table 2 presents a vote-counting synthesis based on the directionality of reported effects. It highlights whether each study reported improvements in mapping accuracy, limitations of the CCEP technique, utility in connectivity mapping, or issues related to data processing. This helps illustrate both strengths and weaknesses observed across studies.

**Table 2:** Vote counting based on directionality of reported effects

Study	Improved mapping accuracy	Highlighted limitations	Connectivity mapping	Data processing impact
Lega et al. (2015) [15]	✔	✖	✔	✖
Shimada et al. (2017) [16]	✖	✔	✔	✔
Prime et al. (2018) [17]	✔	✔	✔	✔
Chirchiglia et al. (2020) [18]	✔	✖	✔	✖
Boyer et al. (2021) [19]	✔	✔	✔	✔
Seidel et al. (2022) [20]	✔	✖	✔	✖
Hays et al. (2023) [21]	✔	✖	✔	✖
Levinson et al. (2024) [22]	✖	✔	✖	✔

Table 3 shows the results of the risk of bias assessment using the ROBINS-I tool. Each included study was evaluated across five domains, including confounding, selection, intervention classification, missing data, and outcome measurement. The overall risk rating is also included to support the interpretation of the reliability of the evidence. 

**Table 3:** Risk of bias assessment using ROBINS-I ROBINS-I: Risk of Bias in Non-randomized Studies of Interventions

Study	Confounding	Selection	Intervention	Missing data	Measurement	Overall risk
Lega et al. (2015) [15]	Low	Moderate	Low	Low	Low	Moderate
Shimada et al. (2017) [16]	Low	Moderate	Moderate	Low	Moderate	Moderate
Prime et al. (2018) [17]	Serious	Serious	Serious	N/A	Serious	Serious
Chirchiglia et al. (2020) [18]	Serious	Moderate	Serious	N/A	Serious	Serious
Boyer et al. (2021) [19]	Low	Low	Low	Low	Moderate	Low
Seidel et al. (2022) [20]	Low	Low	Low	Low	Low	Low
Hays et al. (2023) [21]	Low	Low	Low	Low	Low	Low
Levinson et al. (2024) [22]	Low	Low	Low	Low	Moderate	Low

Table 4 presents a thematic summary of key outcomes grouped into four domains: mapping connectivity, stimulation parameters, signal processing, and clinical utility. Representative studies are listed for each domain, showing the specific contributions of individual papers to thematic insights. 

**Table 4:** Thematic summary of key outcomes CCEP: cortico-cortical evoked potential

Theme	Key observations	Representative studies
Mapping connectivity	CCEPs identify long-range cortical interactions	Lega et al. (2015) [15], Boyer et al. (2021) [19], Hays et al. (2023) [21]
Stimulation parameters	Titration critical; lack of standardization	Prime et al. (2018) [17], Hays et al. (2023) [21]
Signal processing	Preprocessing alters CCEP interpretability	Levinson et al. (2024) [22], Shimada et al. (2017) [16]
Clinical utility	Multimodal integration optimizes mapping	Chirchiglia et al. (2020) [18], Seidel et al. (2022) [20]

Table 5 summarizes the overall effect direction of each study, categorized as positive, mixed, or neutral. It offers a quick visual synthesis of the consistency of CCEP-related benefits across the reviewed literature, supporting the narrative synthesis.

**Table 5:** Summary of effect direction VCP: volume-conducted potential; EP: evoked potential; CCEP: cortico-cortical evoked potential

Study	Effect direction	Summary
Lega et al. (2015) [15]	✅ Positive	Differentiated seizure spread zones
Shimada et al. (2017) [16]	⚠️ Mixed	VCP artifacts complicate signal
Prime et al. (2018) [17]	✅ Positive	Protocol recommendations
Chirchiglia et al. (2020) [18]	❌ Neutral	No primary CCEP application
Boyer et al. (2021) [19]	✅ Positive	Differentiated EP components
Seidel et al. (2022) [20]	✅ Positive	Multimodal intraoperative mapping
Hays et al. (2023) [21]	✅ Positive	Stimulation affects localization
Levinson et al. (2024) [22]	✅ Positive	Preprocessing shapes outcomes

Table 6 provides a descriptive summary of the CCEP approaches used in each study. It includes details on the study population, recording modality (stereo-electroencephalography, SEEG; electrocorticography, ECoG; etc.), purpose of applying CCEP, and the primary findings. This comparative overview helps contextualize how and why CCEPs were used differently depending on clinical goals.

**Table 6:** Descriptive table of CCEP approaches SEEG: stereo-electroencephalography; ECoG: electrocorticography; DES: direct electrical stimulation; EP: evoked potential; CCEP: cortico-cortical evoked potential

Study	Population	Modality	Purpose of CCEP	Key finding
Lega et al. (2015) [15]	Epilepsy (n=37)	SEEG	Seizure mapping	Early spread = stronger signal
Shimada et al. (2017) [16]	Epilepsy (n=8)	ECoG	Modeling volume effects	Volume conduction challenges
Prime et al. (2018) [17]	N/A	Review	Safety and protocol	Best practice insights
Chirchiglia et al. (2020) [18]	N/A	Opinion	Multimodal imaging	Eloquent region targeting
Boyer et al. (2021) [19]	Tumor (n=2)	DES	Awake EP differentiation	Feasible and informative
Seidel et al. (2022) [20]	N/A	Review	Intraop planning	CCEP + imaging strategy
Hays et al. (2023) [21]	Epilepsy (n=15)	SEEG	SOZ localization	Intensity affects CCEP response
Levinson et al. (2024) [22]	Dataset	ECoG	Signal refinement	Referencing alters output

Discussion

This systematic review synthesizes emerging evidence on the clinical utility and methodological nuances of cortico-cortical evoked potential (CCEP) monitoring in brain tumor surgery involving eloquent regions [23]. The consistency in reporting improved mapping accuracy-seen in six of eight studies-underscores CCEP’s translational relevance in neurosurgical practice, particularly in contexts where preserving language, motor, or sensory function is paramount [24].

The reviewed literature suggests that CCEP monitoring adds value as both a diagnostic and intraoperative mapping tool. Some studies demonstrate that modulation of stimulation parameters-especially titration of intensity-directly influences the amplitude and reliability of recorded potentials, which in turn affects the localization of eloquent areas and seizure onset zones. Moreover, the findings support the feasibility of integrating CCEP into awake craniotomies, offering a real-time functional assessment of white matter tracts in relation to tumor margins.

Despite these advances, several methodological limitations were noted [25]. There is a conspicuous lack of standardization in CCEP protocols, including stimulation frequency, pulse duration, and analytic techniques [26]. As highlighted by one of the studies, preprocessing decisions (e.g., referencing schemes and filter settings) significantly alter the amplitude and latency of evoked responses, thereby affecting reproducibility and inter-study comparability. Similarly, a study also raised concerns regarding volume-conducted potentials (VCPs), which may confound interpretations in densely packed cortical areas.

An additional challenge relates to heterogeneity in surgical populations-most studies included patients with epilepsy, not solely brain tumors-limiting the direct generalizability of findings to oncological neurosurgery [27]. Moreover, only a few studies employed rigorous control groups or randomization, and the small sample sizes further restrict external validity. These concerns were reflected in our ROBINS-I assessments, where only three studies had a low overall risk of bias, and most had at least moderate concern in one or more domains [28].

Importantly, several studies advocated multimodal integration, combining CCEP with diffusion tensor imaging (DTI), SEEG, or functional MRI. This aligns with a growing consensus that no single modality is sufficient for safe resection in eloquent cortex, and that CCEP should be viewed as a complementary rather than standalone technique.

Finally, the absence of pooled effect sizes and funnel plot analysis reflects the field’s nascent stage and the need for larger, prospective, and standardized trials. Until such data emerge, CCEP remains a promising but context-dependent tool whose application requires technical expertise and interpretive caution.

## Conclusions

CCEP monitoring represents an increasingly valuable adjunct in the neurophysiological armamentarium for mapping eloquent brain regions during tumor surgery. This review highlights its capacity to inform surgical planning, preserve critical functions, and enhance intraoperative decision-making. However, widespread adoption is currently limited by methodological heterogeneity, inconsistent preprocessing protocols, and a paucity of large-scale clinical studies. Future research should prioritize standardized CCEP methodologies, integrate multimodal mapping approaches, and evaluate clinical outcomes in prospective, tumor-specific cohorts. With further refinement and validation, CCEP holds the potential to significantly improve the safety and precision of neurosurgical interventions in eloquent cortex.
